# Genetic association between interleukin-17 and susceptibility to rheumatoid arthritis

**DOI:** 10.1186/s12920-023-01713-6

**Published:** 2023-11-06

**Authors:** Rong Zhao, Yi-wen Zhang, Jia-yuan Yao, Jun Qiao, Shan Song, Sheng-xiao Zhang, Cai-hong Wang, Xiao-feng Li

**Affiliations:** 1 Department of Rheumatology, Second Hospital of Shanxi Medical University, Taiyuan, 030001 Shanxi China; 2 Key Laboratory of Cellular Physiology at Shanxi Medical University, Ministry of Education, Taiyuan, China

**Keywords:** Rheumatoid arthritis, Interleukin-17, Interleukin-17 receptor, Mendelian randomization, Genome-wide association study

## Abstract

**Background:**

The pathogenesis of rheumatoid arthritis (RA) is an immune imbalance, in which various inflammatory immune cells and pro-inflammatory factors are involved. Interleukin-17 (IL-17), a potent pro-inflammatory cytokine, has been found to have increased expression in the joints of patients with RA compared to healthy individuals. However, the causal relationship between the expression level of IL-17 or IL-17 receptor (IL-17R) and RA remained unknown. In this study, two-sample Mendelian randomization (MR) was used to investigate the causal relationship between IL-17 and RA.

**Methods:**

Summary statistics for RA (14,361 RA cases and 43,923 healthy controls) and IL-17 (3,301 samples) were obtained from an available meta-analysis of published genome-wide association studies (GWAS). Relevant single nucleotide polymorphisms (SNPs) were selected by executing quality control steps from the GWAS summary results. Then we used bi-directional two-sample Mendelian randomization (MR) and multi-variable MR (MVMR) analysis to examine evidence of causality. MR and MVMR analyses progressed mainly using inverse variance weighted (IVW), weighted median (WM), and MR-Egger regression methods, which were applied to the genetic instrumental variables (IVs) of IL-17A/IL-17 RA, IL-17C/IL-17 RC, and IL-17D/IL-17RD and RA. For assessing the robustness of the results, we also carried out a sensitivity analysis to assess heterogeneity and pleiotropy, such as MR-Egger, leave-one-out, and MR pleiotropy residual sum and outlier (MR-PRESSO).

**Results:**

Two-sample MR Analysis showed the causal relationship between IL-17A/IL-17RA and RA. The presence of genetically high IL-17A/IL-17RA may increase the risk of RA (IL-17A(OR = 1.095; 95% C.I., 0.990-1.210, p.adj = 0.013), IL-17RA(OR = 1.113, 95%CI = 1.006-1.231, p.adj = 0.006)). However, the results indicated that IL-17C/IL-17RC, and IL-17D/IL-17RD demonstrated no causal impact on RA (IL-17C(OR = 1.007, 95%CI = 0.890-1.139, p.adj = 0.152), IL-17RC(OR = 1.006, 95%CI = 0.904-1.119, p.adj = 0.152), IL-17D(OR = 0.979, 95%CI = 0.843-1.137, p.adj = 0.130), IL-17RD(OR = 0.983, 95%CI = 0.876-1.104, p.adj = 0.129)). Furthermore, MVMR analysis shown that IL-17RA(OR = 1.049, 95% CI: 0.997-1.102, p.adj = 0.014) was associated with increased risk of RA. Sensitivity analysis showed no heterogeneity and pleiotropy, suggesting that the above results were robust and reliable.

**Conclusion:**

The MR analysis provides evidence that IL-17A/IL-17RA are risk factors for RA. This emphasizes the importance of intervention on IL-17A/IL-17RA in patients with RA. Developing drugs that limit IL-17A may reduce the risk of RA.

**Supplementary Information:**

The online version contains supplementary material available at 10.1186/s12920-023-01713-6.

## Introduction

Rheumatoid arthritis (RA) is a typical systemic autoimmune disease characterized by chronic joint inflammation and bone destruction [1–3]. It comprises symmetric polyarthritis affecting diarthrodial joints and periarticular structures and presenting several systemic manifestations [4]. A variety of inflammatory immune cells and proinflammatory factors are directly involved in the RA process [5–7]. Among them, interleukin-17 (IL-17), a potent pro-inflammatory cytokine, has been found to have increased expression in the joints of RA patients compared to healthy individuals. IL-17 is produced by many types of adaptive and innate immune cells [8], which protects the host from fungal and extracellular bacterial infection and plays an essential role in the pathogenesis of RA [9]. However, the causal relationship between the expression levels of various subtypes of IL-17 or IL-17 receptor (IL-17R) and RA remained unclear.

The IL-17 family consists of six structurally related cytokines, namely IL-17A through IL-17F. The IL-17 receptor family comprises five subunits termed Interleukin-17A receptor (IL-17RA) through IL-17RE [10–12]. Ligation of IL-17R by IL-17 activates the phosphorylation of Mitogen-Activated Protein Kinases (MAPK) and nuclear factor-κB(NF-κB) pathway to induce pro-inflammatory cytokines in tissue homeostasis [13, 14]. Those pro-inflammatory cytokines boost some sites, such as the synovial tissue, for the development of inflammation, making further efforts to promote the occurrence of RA [15]. Although some literature suggests that IL-17 or IL-17R and RA maybe have a connection, it remains unclear whether the causal relationship between them [9]. So, we researched the relationship of those factors based on predecessors by MR.

Mendelian randomization (MR) selected single nucleotide polymorphism (SNP) as an instrumental variable (IV) to test the causal relationship between exposure and outcome by using genetic markers associated with exposure [9]. The advantages of using MR for causal inference are as follows. MR used the genetic variation index to measure the causality of disease-related risk factors, overcoming the bias caused by confounding or reverse causality inherent in observational studies [16]. MR can also evaluate the robustness of causal effect estimates by testing the existence of pleiotropy [17]. Two-sample MR analysis allows the use of aggregate statistics from genome-wide association studies (GWAS) for MR studies without directly analyzing individual-level data [18]. Based on public GWAS data from a large number of people, we use a two-sample MR analysis to illustrate the impact of IL-17 on RA.

## Methods

### Study design

**Fig. 1:**
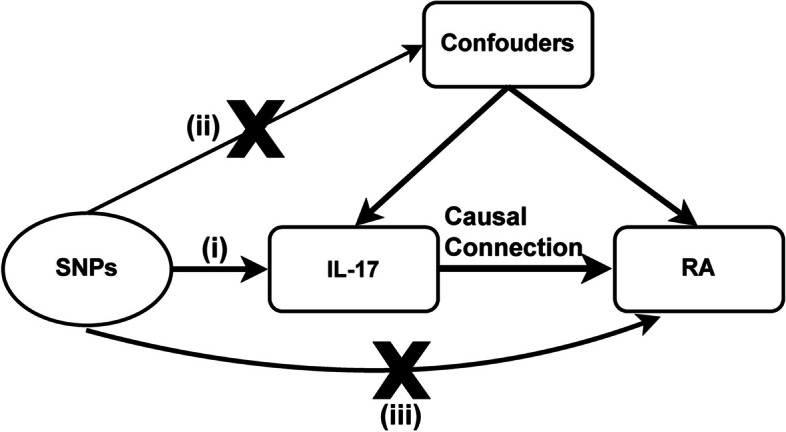
Three basic assumptions in MR analysis. The arrow indicates the causal relationship. *SNP* Single nucleotide polymorphism, *RA* Rheumatoid arthritis

In this study, we conducted a two-sample MR to evaluate the causal association between RA and IL-17. Relevant SNPs were selected by executing quality control steps from the GWAS summary results. To maximize the accuracy of results, the elected SNPs as IVs, which need to meet three basic assumptions (Fig. 1): (i)SNP was strongly associated with exposure (correlation hypothesis); (ii)SNP was not associated with confounding factors, meaning that the results were not affected by confounding factors (independence assumption); (iii)SNP was not associated with the outcome or did not directly affect the outcome (exclusivity hypothesis). All original studies acquired ethical review approval and informed consent.

### Genetic datasets for IL-17

Genetic prediction of the exposure to the IL-17-related gene GWAS data were retrieved from a recent GWAS of the human plasma proteome results from the INTERVAL study with 3301 healthy individuals of European descent (mean age 43.7 years, 51.1% men). The study performed the SomaLogic method to assess 3,622 plasma proteins [19], covering 10,534,735 genotyped SNPs. We applied the IL-17A/ IL-17 RA, IL-17C/ IL-17 RC, and IL-17D/IL-17RD to explore the causal relationship between IL17 and RA.

### Genetic datasets associated with RA

The RA-related outcome data set comes from a previous summary data from GWAS conveyed by Okada et al. [20], including 14,361 cases and 43,923 healthy controls of European descent with a total of 8,747,963 SNPs related to RA. All RA cases fulfilled the 1987 RA diagnosis criteria of the American College of Rheumatology [21] or were diagnosed as RA by a professional rheumatologist.

### Selection of IVs

Several quality control steps were taken in our analysis to select qualified IVs closely associated with IL-17. First, to satisfy the correlation hypothesis, we deleted SNPs with a minor allele frequency (MAF) < 1% to enhance the statistical power of genetic variants. Moreover, we removed variants with a physical distance of less than 10,000 kb and r^2^ < 0.001 to avoid linkage disequilibrium (LD). In the process of selecting independent snps that are closely related to IL-17 and have p-values less than 5e-8, we found that 1 SNP was associated with IL-17A, IL-17RC and IL-17RD, 3 SNPs were connected with IL-17RA, none SNP were associated with IL-17C and IL-17D. In order to satisfy enough eligible IVs for subsequent studies, we broadened the threshold to 1e-5. We also analyzed whether these SNPs were possible confounding factors in PhenoScanner (http://www.phenoscanner.medschl.cam.ac.uk/) to satisfy the independence hypothesis, such as smoking, alcohol consumption, body mass index, and SNPs associated with these potential aspects were excluded. Finally, to guarantee that the effect of a SNP on the exposure and the effect of that SNP on the outcome must each correspond to the same allele, we harmonized the exposure and outcome datasets by correct the strand for non-palindromic SNPs and drop all palindromic SNPs. We utilized these carefully chosen SNPs as the final genetic IVs for the subsequent MR analysis.

The F statistic is used to test the correlation hypothesis that SNP was strongly associated with exposure, calculated using the formula: $\mathrm{F}=\frac{{\mathrm{R}}^{2}\left(\mathrm{n}-\mathrm{k}-1\right)}{\left(1-{\mathrm{R}}^{2}\right)\mathrm{k}}$. R^2^ denotes the variance of exposure explained by IVs, n is the sample size and k is the number of instrumental variables [22]. For Mendelian randomization, the F statistic is an indicator of the strength of the IVs, with values over 10 reflecting strong instruments. If the F statistic less than ten, IVs were considered weak instruments and would be excluded for MR analysis [23].

### Statistical analysis

We utilized these MR methods from TwoSampleMR packages in R software (version 4.1.0) to estimate the causal effects of the cytokines and cytokine receptors (A, C, D) of IL-17 on RA. We used inverse variance weighted (IVW), weighted median (WM), and MR-Egger regression methods to assess the causal influence of the exposure on the outcome [24–26]. IVW is the primary analysis method that can balance pleiotropy. MR-egger and weighted median enhance the estimation of IVW, although less efficiently [27].

### Sensitivity analysis

To assess the robustness of these results and prevent potential pleiotropy and heterogeneity, a series of sensitivity analyses, including MR-Egger intercept tests, MR-pleiotropy residual sum and outlier (MR-PRESSO) global test, and Cochran's Q test for heterogeneity, Funnel pot test, leave-one-out analyses. A *P* value of < 0.05 in the Cochran Q test was considered statistically significant. MR-egger was used to evaluate the potential horizontal pleiotropy. The intercept term of MR-Egger regression was used to assess whether horizontal pleiotropy affected the results of MR analysis, where *P* < 0.05 indicated horizontal pleiotropy [28]. By visual inspection of funnel plots, asymmetries indicate horizontal pleiotropy. We performed a Leave-one-out analysis to determine whether any single SNP drove the causal estimates. We repeated the IVW analysis by discarding each exposure-related SNP [24]. In the MR-PRESSO test, SNPs associated with heterogeneity were eliminated to reduce outliers in the estimation of causal effects [29].

Bonferroni correction method was used to account for multiple testing in this study. Associations with adjust *p* value (p.adj) < 0.05 were regarded as significant associations. Since this MR study was conducted using publicly available GWAS summary data, ethical approval and informed consent from all subjects could be found in the original publications.

## Result

### IVs for MR

The selection process of IL-17RA-related SNPs is detailed below, and other exposure-related SNPs are shown in Table 1. A total of 25 SNPs associated with IL-17RA were identified after the genome-wide significance threshold (*p* < 1 × 10^–5^) and clumping (r^2^ < 0.001). Among them, 6 SNPs were correlated with outcome RA after the harmonizing process, and none of them was an outlier variant detected by the MR-PRESSO test. We checked in PhenoScanner V2 whether the previously retained SNPs were connected with confounding factors, such as smoking, drinking, and body mass index (BMI), and there were no abnormal SNPs. Finally, 6 SNPs were chosen as instrumental variables for IL-17RA in the present study.

**Table 1:** Associations of IL-17 levels with rheumatoid arthritis in MR with IVW, MR-Egger, WM method

Outcomes	SNPs	MR methods	OR	95%CI	pval	Q_pval	p.adj
IL-17A	12	IVW	1.095	0.990, 1.210	0.078	0.701	0.013*
		MR-Egger	0.844	0.540, 1.320	0.475	0.747	0.079
		WM	1.135	0.994, 1.297	0.063	-	0.011*
IL-17RA	6	IVW	1.113	1.006, 1.231	0.038	0.763	0.006*
		MR-Egger	1.209	0.934, 1.564	0.223	0.714	0.037*
		WM	1.142	1.008, 1.293	0.034	-	0.006*
IL-17C	9	IVW	1.007	0.890, 1.139	0.910	0.594	0.152
		MR-Egger	1.139	0.884, 1.468	0.348	0.625	0.058
		WM	1.073	0.908, 1.268	0.414	-	0.069
IL-17RC	13	IVW	1.006	0.904, 1.119	0.913	0.084	0.152
		MR-Egger	1.095	0.703, 1.706	0.696	0.063	0.116
		WM	0.934	0.822, 1.061	0.283	-	0.047*
IL-17D	10	IVW	0.979	0.843, 1.137	0.778	0.189	0.130
		MR-Egger	0.882	0.599, 1.300	0.545	0.154	0.091
		WM	0.981	0.812, 1.184	0.842	-	0.140
IL-17RD	11	IVW	0.983	0.876, 1.104	0.773	0.61	0.129
		MR-Egger	0.903	0.583, 1.396	0.656	0.53	0.109
		WM	0.949	0.812, 1.109	0.526	-	0.088

*indicates *P* < 0.05

**Fig. 2:**
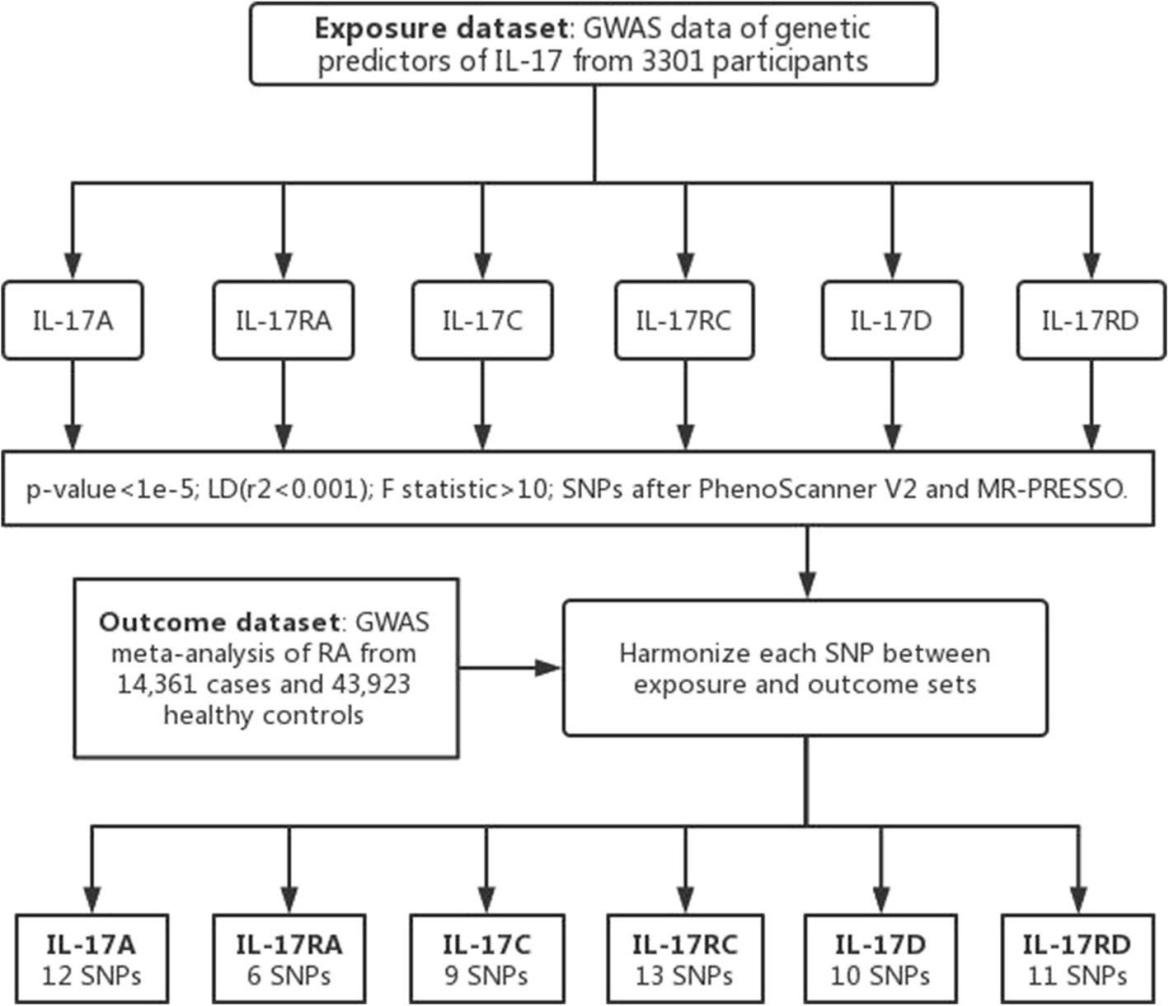
The selection process of instrumental variables for MR analysis

After the same quality control steps, 12 independent SNPs were related to IL-17A, 9 independent SNPs were associated with IL-17C, 13 independent SNPs were connected with IL-17RC, 10 independent SNPs were related with IL-17D, 11 independent SNPs were correlated with IL-17RD. Among the SNPs connected with IL-17RD, rs6776722 (associated with alcohol) and rs400824 (associated with BMI) were excluded. The selection process of IVs for MR analysis is shown in Fig. 2 and these SNPs are shown in detail in Supplementary Table 1.

### Mendelian randomization results

**Fig. 3:**
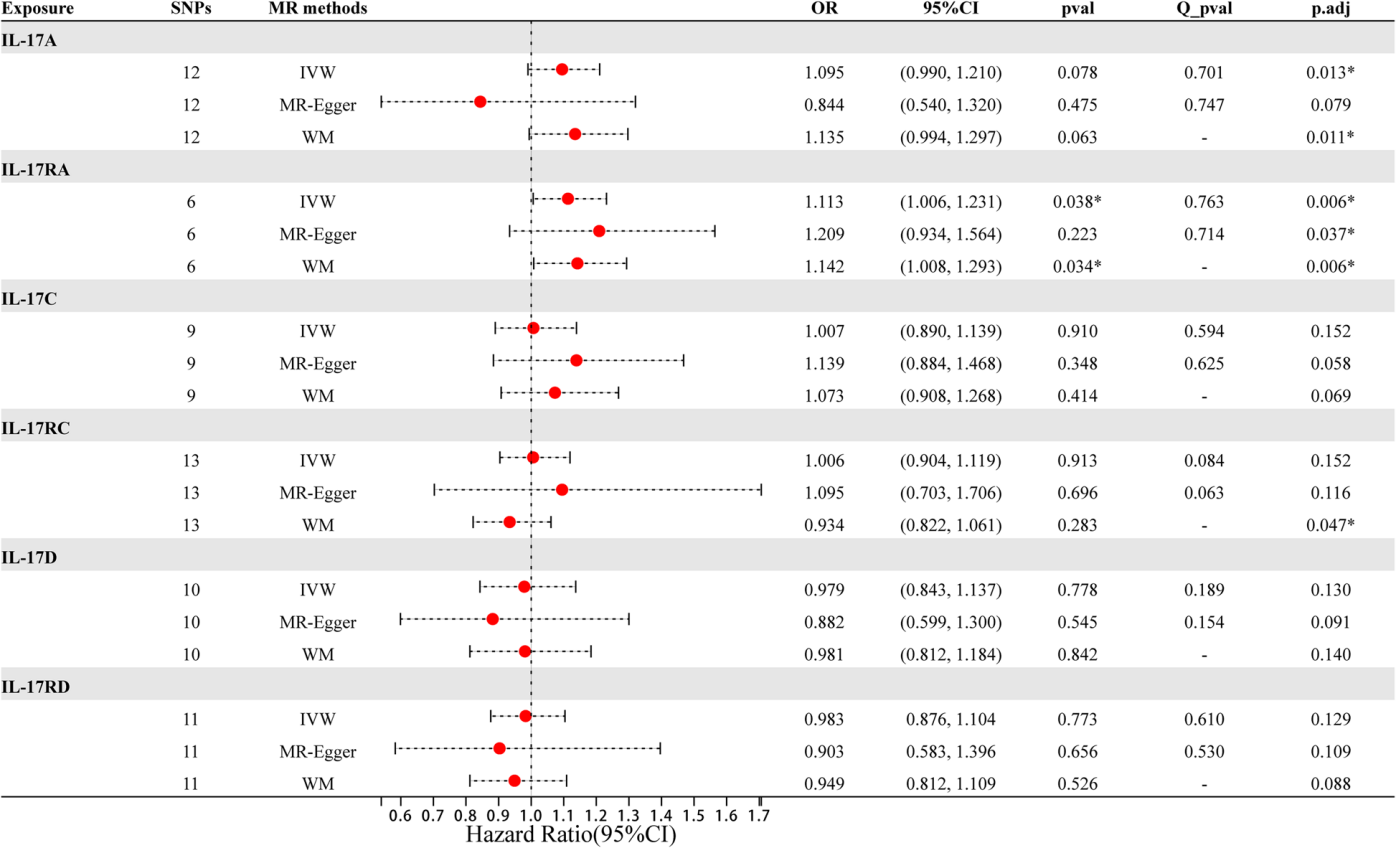
Forest plot of MR results by IVW, MR Egger, and weighted median

**Fig. 4:**
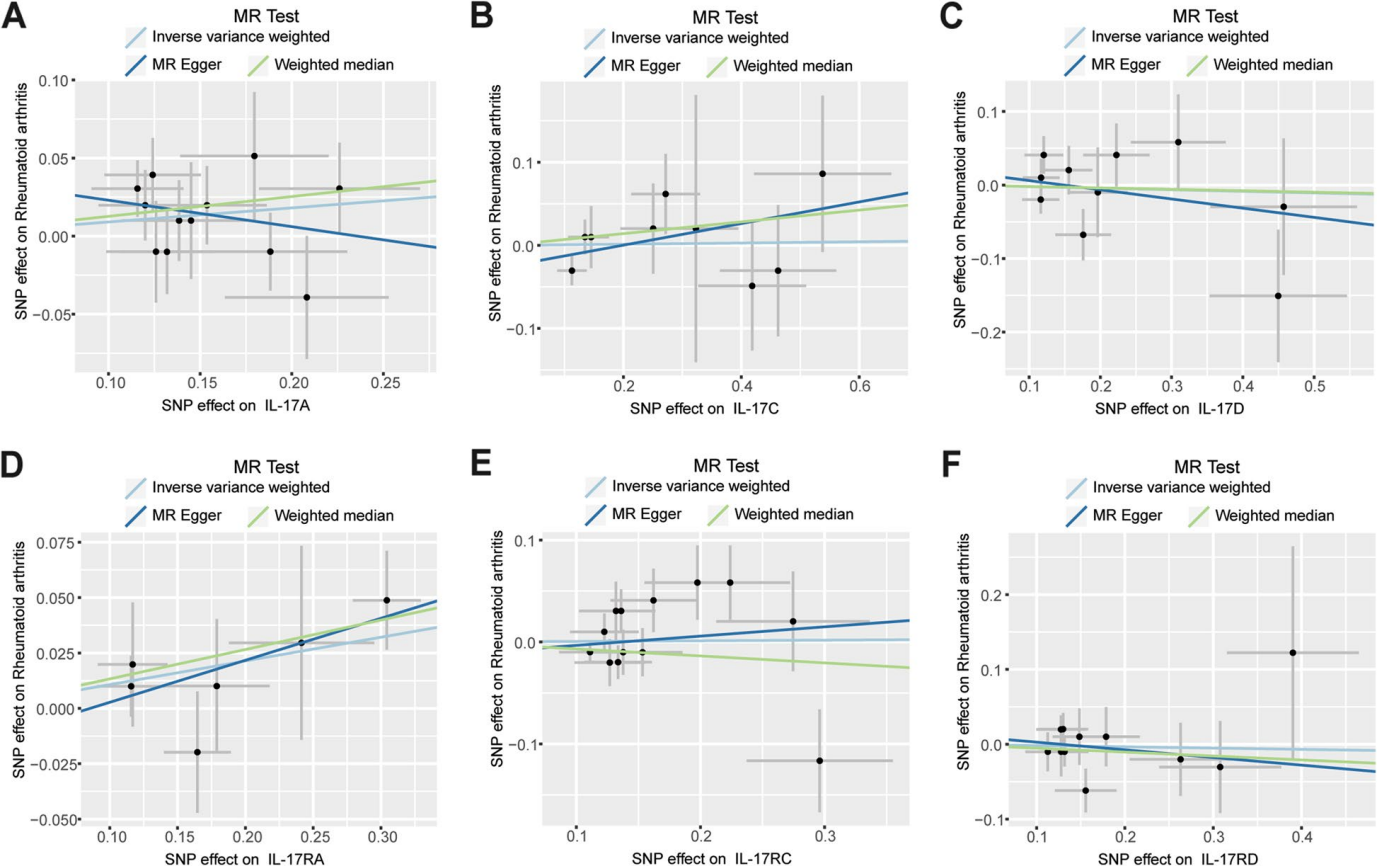
The scatter plots for MR analyze the causal effect between IL-17 and RA using the conventional IVW, MR-Egger, and Weighted median. Scatter plots showed the effect of IL-17A (**A**), IL-17C (**B**), IL-17D (**C**), IL-17RA (**D**), IL-17RC (**E**) and IL-17RD (**F**) on RA

We evaluated the causal effect of IL-17A/IL-17RA, IL-17C/IL-17RC, and IL-17D/IL-17RD on RA using two-sample MR methods (Table 1). After Bonferroni-correction, according to the IVW and weighted-median analysis, the presence of genetically high IL-17A/IL-17RA were associated with increased risk of RA (IL-17A(OR = 1.095; 95% C.I., 0.990–1.210, p.adj = 0.013), IL-17RA(OR = 1.113, 95%CI = 1.006–1.231, p.adj = 0.006)).. In addition, IVW analyses showed that IL-17C/IL-17RC, and IL-17D/IL-17RD demonstrated no causal impact on RA (IL-17C(OR = 1.007, 95%CI = 0.890–1.139, p.adj = 0.152), IL-17RC(OR = 1.006, 95%CI = 0.904–1.119, p.adj = 0.152), IL-17D(OR = 0.979, 95%CI = 0.843–1.137, p.adj = 0.130), IL-17RD(OR = 0.983, 95%CI = 0.876–1.104, p.adj = 0.129)), and these results were confirmed by the weighted-median and MR-Egger analyses. Forest plots and scatter plots of the association between IL-17A/IL-17RA, IL-17C/IL-17RC, and IL-17D/IL-17RD and RA are shown in Fig. 3 and Fig. 4.

### Sensitivity analysis

**Fig. 5:**
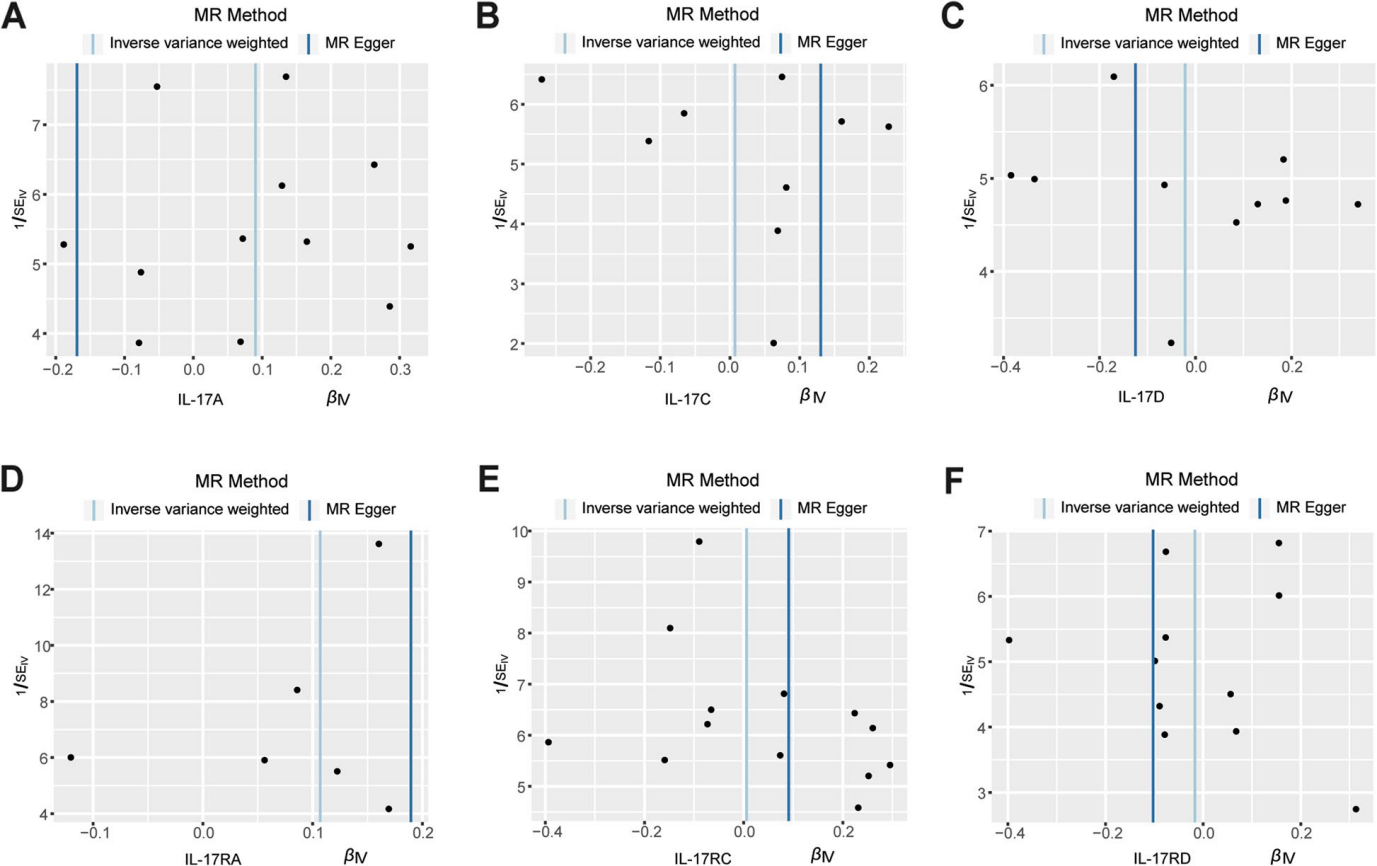
IVW and MR-Egger regression slopes were used to explore asymmetry as a sign of pleiotropy, with the vertical line in the middle indicating the sum of different effect sizes. Funnel plot of IL-17A (**A**), IL-17C (**B**), IL-17D (**C**), IL-17RA (**D**), IL-17RC (**E**) and IL-17RD (**F**) genetic liability effects on RA

**Fig. 6:**
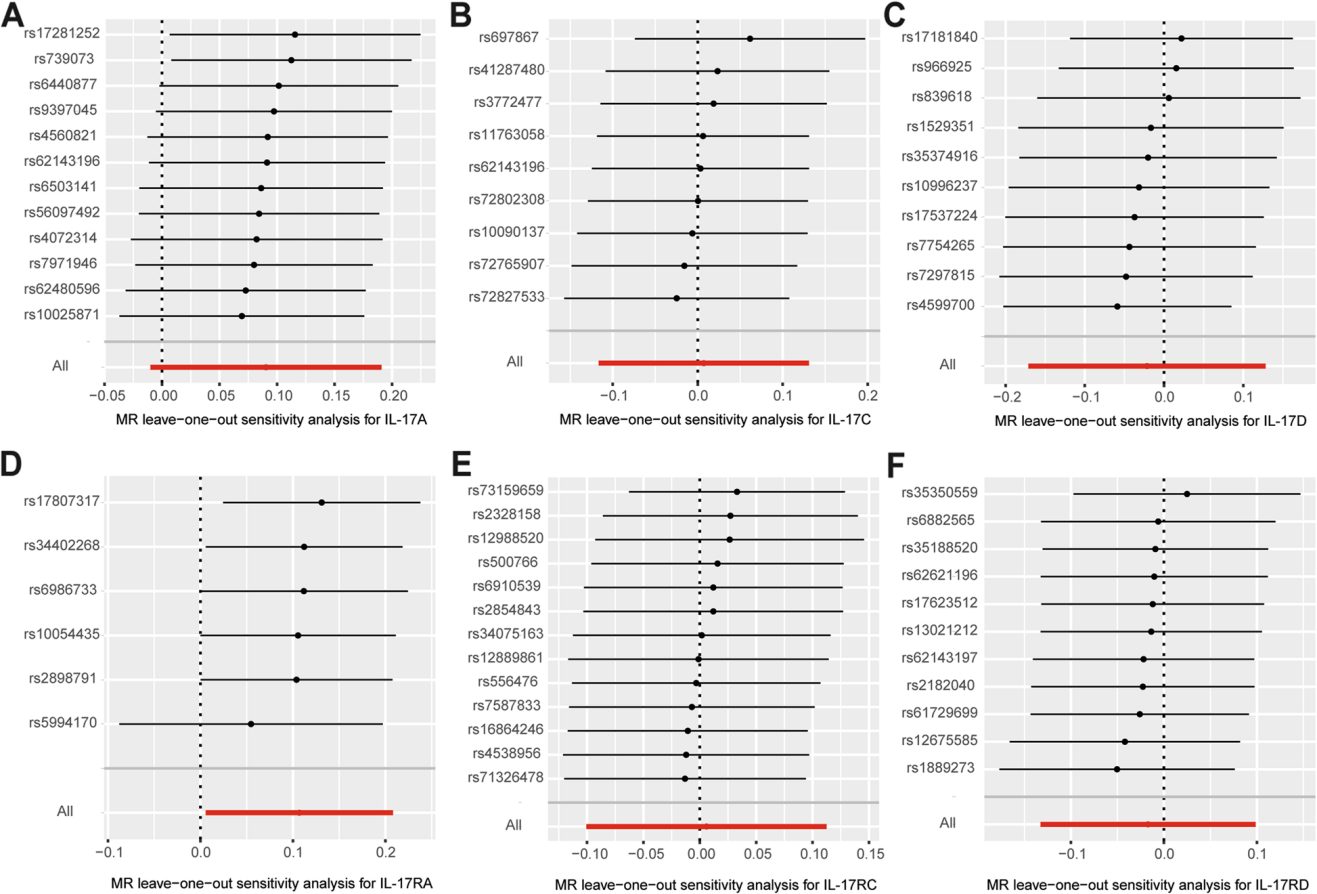
Leave-one-out analysis was used to determine whether any single SNP drove the causal association of IL-17A (**A**), IL-17C (**B**), IL-17D (**C**), IL-17RA (**D**), IL-17RC (**E**) and IL-17RD (**F**) on RA, which repeated the IVW analysis by discarding each exposure-related SNP

Regarding heterogeneity and sensitivity, Cochran’s Q-test (P_Q_ > 0.05) and MR-Egger regression indicated no heterogeneity in the causal effect between IL-17A/ IL-17 RA, IL-17C/ IL-17 RC, IL-17D/IL-17RD, and RA. The funnel plot displayed a symmetric pattern of effect size variation around the point estimate (Fig. 5). In the leave-one-out analyses, we found that the risk estimates of IL-17 levels and risk of RA kept consistent substantially after excluding one SNP at each time, which means IL-17-RA association was not driven by any individual SNP exclusion, suggesting that SNPs without potential influence causal relationship and the conclusion was stable and reliable (Fig. 6). As for pleiotropic analysis, the pooled causal effects of the MR-Egger regression analysis were consistent with the IVW results, indicating that all variables were effective for RA. MR-PRESSO indicated no presence of potential pleiotropy in our tests.

### Multivariable MR analysis

In model with mutual adjustment for IL17A, IL17C, IL17D, IL17RA, IL17RC and IL17RD, the association between IL17RA and risk of RA was significant (nSNP = 52, OR = 1.049, 95% CI: 0.997–1.102, p.adj = 0.014, Table2). This finding again suggested the IL17RA is one of risk factor for RA.

**Table 2:** The associations of IL-17 cytokines with RA in multivariable MR with inverse-variance weighted method

Exposure	Outcome	nSNP	Beta	SE	OR (95%CI)	*P*-value	P.adj
IL17A	RA	52	0.041	0.062	1.064(1.011–1.118)	0.515	0.086
IL17C	RA	52	0.047	0.067	1.069(1.016–1.122)	0.481	0.080
IL17D	RA	52	-0.047	0.064	1.066(1.012–1.119)	0.456	0.076
IL17RA	RA	52	0.083	0.048	1.049(0.997–1.102)	0.083	0.014*
IL17RC	RA	52	-0.039	0.053	1.054(1.001–1.107)	0.460	0.077
IL17RD	RA	52	-0.019	0.039	1.040(0.988–1.092)	0.624	0.104

*indicates *P* < 0.05

## Discussion

This study used MR analyses firstly to investigate potential causal links between a set of IL-17 family and RA, which found that IL-17A/IL-17RA maybe contribute to the higher risk of RA. However, there were no causal effects of IL-17C/ IL-17RC, and IL-17D/ IL-17RD on RA according to the MR results. These findings would contribute greatly to research on the mechanism and treatment of RA.

Studies in the 1990s showed that compared to healthy individuals, IL-17 expression had increased in the joint of RA patients [30, 31]. And high levels of serum IL-17 A have been reported in RA [32]. In addition, IL-17RA were over-expressed in RA peripheral blood, and their expression was detected locally in RA synovium [33]. IL-17A is involved in inflammation and defense against infection by inducing fibroblasts, endothelial cells, and epithelial cells to express pro-inflammatory cytokines (TNF, IL-1, IL-6, G-CSF, and GM-CSF), chemokines (CXCL1, CXCL5, IL-8, CCL2, and CCL7), and antimicrobial peptides (defensin and S100 protein) and matrix metalloproteinases (MMP1, MMP3, and MMP13) [11, 34, 35]. IL-17A promotes granulocyte formation mediated by stem cell factor and granulocyte colony-stimulating factor and recruits neutrophils to inflammatory sites [31, 32, 36]. IL-17A induces keratinocytes to express intercellular adhesion molecule-1 (ICAM-1) and chondrocytes to express iNOS and cyclooxygenase-2 [37, 38]. These may explain why IL-17A/IL-17RA is a risk factor for RA. However, we found no direct causal relationship between IL-17C/IL-17RC, and IL-17D/IL-17RD and RA. They may participate in the occurrence and development of RA through other risk factors or pathways.

Drugs that restrict IL-17A may reduce the risk of RA. Currently, monoclonal antibodies (Mabs) targeting IL-17 or IL-17R include Ixekizumab, Secukinumab, Brodalumab, and bimekizumab. Ixekizumab (Taltz®, LY2439821) is a humanized IgG4 monoclonal antibody [39]. Secukinumab (AIN457, Cosentyx) is a recombinant human IgG1/kappa monoclonal antibody [40, 41]. Brodalumab (AMG 2) is a fully human IgG17 monoclonal antibody [42]. Bimekizumab (496.g3, formerly UCB4940) is a humanized monoclonal antibody with a high affinity for IL-17A and IL-17F [43]. They all target IL-17A and inhibit its binding and interaction with its receptors, such as blocking IL-17A, IL-17F, and IL-17-A/F heterodimer signaling and IL-17E signaling in IL-17RA/RC complexes. The secretion of protein kinases, pro-inflammatory cytokines, and chemokines is then inhibited by targeting cells with downstream effects on cellular elements [8].

There are several advantages to our MR analysis. Compared with randomized controlled trials, when selecting appropriate tool variables, this method reduced the interference of confounding factors to a greater extent. In our study, we excluded the influence of horizontal pleiotropy, further indicating the study's reliability. Additionally, we analyzed all IL-17 and IL17RA one by one, which highly improved universality and expansibility. Even so, there are several limitations of the study. For example, the summary GWAS data we mainly used refers to individuals of European ancestry, so it should be considered accurate when the results are applied to other races. GWAS are able to identify common variants with moderate to small effect sizes, but they may not be sensitive to identifying genes with disease-causing mutations. In addition, we could not further explore the MR effect estimates between IL-17 levels and disease severity due to the lack of relevant data. Moreover, the possible causal relationship between IL-17RA and RA found in the current study will require experimental verifications in a laboratory animal model(s) with careful controlled randomized study design.

## Conclusion

In conclusion, the MR analysis supported a causal relationship between genetically predicted IL-17RA and RA. This study provided strong evidence that IL-17RA is a risk factor for RA. What's more, it will promote studies on reducing the risk of RA by developing drugs that limit IL-17A.

### Supplementary Information

**Additional file 1: Supplementary table 1. **Summary of the 61 SNPs associated with IL-17.

## Data Availability

All data generated or analyzed during this study are included in this published article [Supplementary Table 1].

## Abbreviations

RA: Rheumatoid arthritis
IL-17: Interleukin-17
IL-17RA: Interleukin-17A receptor
MAPK: Mitogen-Activated Protein Kinases
NF-κB: Nuclear factor-κB
MR: Mendelian randomization
SNP: Single nucleotide polymorphism
IV: Instrumental variable
GWAS: Genome-wide association study
MAF: Minor allele frequency
LD: Linkage disequilibrium
IVW: Inverse variance weighted
WM: Weighted median
MR-PRESSO: MR-pleiotropy residual sum and outlier
BMI: Body mass index
Mabs: Monoclonal antibodies
OR: Odds ratio
CI: Confidence interval
