# Supplementary material for: Genetic association between interleukin-17 and susceptibility to rheumatoid arthritis
Source: BMC Med Genomics. 2023 Nov 6;16:277. doi: 10.1186/s12920-023-01713-6 (PMC10626638; doi:10.1186/s12920-023-01713-6)
Supplement: Supplementary file 1 — Additional file 1: Supplementary table 1. Summary of the 61 SNPs associated with IL-17. [file 12920_2023_1713_MOESM1_ESM.docx]

Supplementary table 1: Summary of the 61 SNPs associated with IL-17

|  |  |  |  |  |  |  |  | IL-17(exposure) | | | | RA(outcome) | | |
| --- | --- | --- | --- | --- | --- | --- | --- | --- | --- | --- | --- | --- | --- | --- |
| IL-17 | SNP | Chr | Position | Effect allele | Other allele | R² | F-statistic | EAF | Beta | SE | P value | Beta | SE | P value |
| IL-17A | rs10025871 | 4 | 83705778 | C | T | 0.00647081 | 21.48623654 | 0.59341 | 0.1158 | 0.0251 | 3.89E-06 | 0.0304592 | 0.0180216 | 0.0909997 |
| IL-17A | rs17281252 | 2 | 207921041 | C | T | 0.006084747 | 20.19646968 | 0.09479 | 0.1883 | 0.042 | 7.24E-06 | -0.00995033 | 0.0249472 | 0.69 |
| IL-17A | rs4072314 | 3 | 34059913 | A | G | 0.008389695 | 27.91177472 | 0.09028 | -0.226 | 0.0439 | 2.69E-07 | -0.0304592 | 0.0293885 | 0.3 |
| IL-17A | rs4560821 | 8 | 72290326 | G | A | 0.006301616 | 20.92086807 | 0.20718 | -0.1385 | 0.0311 | 8.71E-06 | -0.00995033 | 0.0258235 | 0.7 |
| IL-17A | rs56097492 | 10 | 98182762 | A | G | 0.006677761 | 22.17803151 | 0.36534 | 0.12 | 0.0255 | 2.57E-06 | 0.0198026 | 0.0225569 | 0.38 |
| IL-17A | rs62143196 | 19 | 54320636 | G | A | 0.007310228 | 24.29403577 | 0.22404 | -0.145 | 0.0303 | 1.74E-06 | -0.00995033 | 0.0373636 | 0.79 |
| IL-17A | rs62480596 | 7 | 128882592 | C | A | 0.007165749 | 23.81042399 | 0.63175 | -0.1241 | 0.0264 | 2.57E-06 | -0.0392207 | 0.023633 | 0.0969996 |
| IL-17A | rs6440877 | 3 | 153121917 | A | C | 0.008459101 | 28.14465175 | 0.583 | 0.1319 | 0.0255 | 2.34E-07 | -0.0100503 | 0.0270275 | 0.709999 |
| IL-17A | rs6503141 | 17 | 8612795 | G | A | 0.006926123 | 23.00863932 | 0.82157 | -0.1537 | 0.0326 | 2.40E-06 | -0.0198026 | 0.0250923 | 0.43 |
| IL-17A | rs739073 | 22 | 33559230 | T | G | 0.007367053 | 24.48428503 | 0.09377 | -0.2082 | 0.0447 | 3.24E-06 | 0.0392207 | 0.0394393 | 0.32 |
| IL-17A | rs7971946 | 12 | 118432017 | A | G | 0.005959333 | 19.7777024 | 0.10298 | -0.1796 | 0.0405 | 9.33E-06 | -0.0512933 | 0.0409179 | 0.21 |
| IL-17A | rs9397045 | 6 | 151629020 | A | G | 0.006503026 | 21.59390796 | 0.28818 | -0.1259 | 0.0272 | 3.72E-06 | 0.00995033 | 0.0325727 | 0.760001 |
| IL-17RA | rs10054435 | 5 | 25569643 | G | T | 0.006505561 | 21.60238097 | 0.05934 | -0.2414 | 0.0536 | 6.76E-06 | -0.0295588 | 0.0438239 | 0.5 |
| IL-17RA | rs17807317 | 22 | 17680519 | C | A | 0.013546252 | 45.30276835 | 0.49754 | 0.1646 | 0.0247 | 2.57E-11 | -0.0198026 | 0.0274093 | 0.47 |
| IL-17RA | rs2898791 | 1 | 4334601 | A | C | 0.006303066 | 20.92570953 | 0.63922 | 0.1169 | 0.0257 | 5.25E-06 | 0.0198026 | 0.028037 | 0.48 |
| IL-17RA | rs34402268 | 9 | 33122212 | A | C | 0.006542242 | 21.72498552 | 0.11556 | -0.1789 | 0.039 | 4.37E-06 | -0.0100503 | 0.0302855 | 0.74 |
| IL-17RA | rs5994170 | 22 | 17615213 | G | A | 0.044104568 | 152.2143175 | 0.39114 | -0.3043 | 0.025 | 5.25E-34 | -0.0487902 | 0.0223451 | 0.0290001 |
| IL-17RA | rs6986733 | 8 | 63438745 | A | G | 0.006561194 | 21.78833777 | 0.57318 | 0.1158 | 0.025 | 3.72E-06 | 0.00995033 | 0.0137725 | 0.47 |
| IL-17C | rs10090137 | 8 | 119173256 | A | C | 0.007623419 | 25.34285986 | 0.3002 | -0.1347 | 0.0286 | 2.57E-06 | -0.0100503 | 0.0208631 | 0.630001 |
| IL-17C | rs11763058 | 7 | 109542915 | G | A | 0.006654363 | 22.09980424 | 0.033 | 0.3229 | 0.072 | 7.41E-06 | 0.0202027 | 0.160771 | 0.9 |
| IL-17C | rs3772477 | 3 | 59957938 | C | T | 0.00688269 | 22.86335669 | 0.01637 | -0.4623 | 0.0986 | 2.75E-06 | 0.0304592 | 0.079049 | 0.7 |
| IL-17C | rs41287480 | 10 | 104837731 | A | G | 0.00674139 | 22.39078964 | 0.01965 | -0.4183 | 0.0918 | 5.13E-06 | 0.0487902 | 0.0776906 | 0.53 |
| IL-17C | rs62143196 | 19 | 54320636 | G | A | 0.007330408 | 24.36159526 | 0.22404 | -0.1452 | 0.0303 | 1.66E-06 | -0.00995033 | 0.0373636 | 0.79 |
| IL-17C | rs697867 | 12 | 116280085 | A | G | 0.006271834 | 20.8213677 | 0.5557 | 0.1127 | 0.0251 | 7.08E-06 | -0.0304592 | 0.0175705 | 0.0830004 |
| IL-17C | rs72765907 | 9 | 124677431 | C | T | 0.006818671 | 22.64923394 | 0.01193 | -0.5378 | 0.1165 | 3.89E-06 | -0.0861777 | 0.0941457 | 0.36 |
| IL-17C | rs72802308 | 2 | 40878101 | A | G | 0.006251348 | 20.75293204 | 0.05262 | -0.2504 | 0.0556 | 6.76E-06 | -0.0202027 | 0.0543294 | 0.709999 |
| IL-17C | rs72827533 | 2 | 71761503 | T | C | 0.006650914 | 22.08827139 | 0.04732 | -0.2716 | 0.0583 | 3.24E-06 | -0.0618754 | 0.0482816 | 0.2 |
| IL-17RC | rs12889861 | 14 | 47459343 | G | A | 0.006457767 | 21.44264463 | 0.31337 | -0.1225 | 0.0276 | 8.91E-06 | -0.00995033 | 0.0179809 | 0.58 |
| IL-17RC | rs12988520 | 2 | 234607394 | C | A | 0.006088537 | 20.20912904 | 0.55404 | 0.111 | 0.025 | 8.71E-06 | -0.00995033 | 0.0113343 | 0.38 |
| IL-17RC | rs16864246 | 2 | 177559549 | A | G | 0.006733651 | 22.36491142 | 0.09542 | 0.1975 | 0.0426 | 3.47E-06 | 0.0582689 | 0.0364592 | 0.11 |
| IL-17RC | rs2328158 | 6 | 141302888 | G | A | 0.007583168 | 25.20802893 | 0.69384 | 0.1336 | 0.0272 | 8.91E-07 | -0.0198026 | 0.0164973 | 0.23 |
| IL-17RC | rs2854843 | 7 | 45931135 | C | T | 0.007123339 | 23.66849373 | 0.18624 | -0.1533 | 0.0322 | 1.95E-06 | 0.0100503 | 0.0235841 | 0.67 |
| IL-17RC | rs34075163 | 11 | 129891077 | A | G | 0.00591725 | 19.63720535 | 0.04091 | -0.2746 | 0.0619 | 9.12E-06 | -0.0202027 | 0.0489806 | 0.68 |
| IL-17RC | rs4538956 | 9 | 124625023 | T | G | 0.007795119 | 25.91813399 | 0.30104 | -0.1361 | 0.0276 | 7.94E-07 | -0.0304592 | 0.0211591 | 0.15 |
| IL-17RC | rs500766 | 10 | 6550590 | T | C | 0.006246877 | 20.73799557 | 0.26327 | 0.1269 | 0.0285 | 8.71E-06 | -0.0202027 | 0.0230126 | 0.38 |
| IL-17RC | rs556476 | 10 | 98539578 | G | A | 0.006345381 | 21.06709201 | 0.76007 | 0.1319 | 0.0298 | 9.33E-06 | 0.0304592 | 0.0287861 | 0.29 |
| IL-17RC | rs6910539 | 6 | 124654962 | A | C | 0.006318791 | 20.97824747 | 0.21125 | 0.1377 | 0.0302 | 5.13E-06 | -0.0100503 | 0.0221489 | 0.649999 |
| IL-17RC | rs71326478 | 3 | 81649406 | A | G | 0.006645884 | 22.07145579 | 0.07138 | 0.2239 | 0.0483 | 3.63E-06 | 0.0582689 | 0.0364592 | 0.11 |
| IL-17RC | rs73159659 | 7 | 129405439 | C | T | 0.008294417 | 27.59214269 | 0.04978 | -0.2961 | 0.0589 | 5.01E-07 | 0.116534 | 0.0504916 | 0.021 |
| IL-17RC | rs7587833 | 2 | 103617979 | T | G | 0.006955779 | 23.10784729 | 0.84299 | -0.1621 | 0.0347 | 2.88E-06 | -0.040822 | 0.0311481 | 0.19 |
| IL-17D | rs10996237 | 10 | 66909326 | T | C | 0.006725564 | 22.33786904 | 0.43736 | 0.1169 | 0.0259 | 6.31E-06 | 0.00995033 | 0.0258235 | 0.7 |
| IL-17D | rs1529351 | 8 | 18907935 | C | T | 0.006686064 | 22.20579345 | 0.01625 | 0.4573 | 0.1029 | 8.91E-06 | -0.0295588 | 0.0927657 | 0.75 |
| IL-17D | rs17181840 | 12 | 67051225 | C | T | 0.006202317 | 20.58914382 | 0.11285 | 0.176 | 0.0391 | 6.92E-06 | -0.0676586 | 0.0349671 | 0.0530005 |
| IL-17D | rs17537224 | 1 | 167587813 | G | T | 0.00692569 | 23.0071934 | 0.17265 | 0.1557 | 0.0331 | 2.63E-06 | 0.0202027 | 0.0329672 | 0.54 |
| IL-17D | rs35374916 | 12 | 45242620 | C | A | 0.006844336 | 22.73507117 | 0.09829 | 0.1965 | 0.042 | 2.95E-06 | -0.00995033 | 0.0607994 | 0.87 |
| IL-17D | rs4599700 | 7 | 67462843 | A | G | 0.005838184 | 19.37327387 | 0.72203 | -0.1206 | 0.0272 | 9.12E-06 | -0.040822 | 0.0255426 | 0.11 |
| IL-17D | rs7297815 | 12 | 118401155 | T | C | 0.007028988 | 23.35277942 | 0.07683 | -0.2226 | 0.0465 | 1.70E-06 | -0.040822 | 0.0427829 | 0.34 |
| IL-17D | rs7754265 | 6 | 17775070 | C | T | 0.006646832 | 22.07462438 | 0.03599 | -0.3095 | 0.0666 | 3.39E-06 | -0.0582689 | 0.0649979 | 0.37 |
| IL-17D | rs839618 | 1 | 120270181 | T | C | 0.006486048 | 21.53716481 | 0.60317 | -0.1164 | 0.0262 | 8.51E-06 | 0.0198026 | 0.0191065 | 0.3 |
| IL-17D | rs966925 | 4 | 41742562 | T | C | 0.007391426 | 24.56589135 | 0.01863 | 0.4496 | 0.0964 | 3.16E-06 | -0.150823 | 0.0900615 | 0.0940005 |
| IL-17RD | rs12675585 | 8 | 31922940 | G | A | 0.006634197 | 22.03238464 | 0.26822 | 0.13 | 0.0284 | 4.90E-06 | 0.0202027 | 0.0216167 | 0.35 |
| IL-17RD | rs13021212 | 2 | 42711119 | T | C | 0.006580942 | 21.85435104 | 0.27969 | 0.1278 | 0.0274 | 3.02E-06 | -0.0100503 | 0.0329001 | 0.760001 |
| IL-17RD | rs17623512 | 17 | 1026163 | T | C | 0.006016505 | 19.96858993 | 0.38531 | 0.1127 | 0.0254 | 9.55E-06 | -0.0100503 | 0.0260831 | 0.7 |
| IL-17RD | rs1889273 | 9 | 122571381 | C | T | 0.006467051 | 21.47367359 | 0.27328 | -0.1276 | 0.028 | 5.25E-06 | -0.0198026 | 0.0187149 | 0.29 |
| IL-17RD | rs2182040 | 13 | 55573019 | G | A | 0.006588905 | 21.88097033 | 0.11681 | 0.1787 | 0.0382 | 2.95E-06 | 0.0100503 | 0.0396702 | 0.8 |
| IL-17RD | rs35188520 | 13 | 96236413 | T | C | 0.006149591 | 20.41303203 | 0.03356 | 0.3079 | 0.0692 | 8.71E-06 | -0.0304592 | 0.0614282 | 0.62 |
| IL-17RD | rs35350559 | 8 | 58970429 | A | G | 0.00650505 | 21.60067243 | 0.15991 | 0.1556 | 0.0348 | 7.94E-06 | -0.0618754 | 0.0291855 | 0.0340001 |
| IL-17RD | rs61729699 | 11 | 6568667 | C | T | 0.008459967 | 28.14755783 | 0.0286 | -0.3902 | 0.0747 | 1.78E-07 | -0.122218 | 0.142177 | 0.39 |
| IL-17RD | rs62143197 | 19 | 54320716 | A | G | 0.007644718 | 25.41420919 | 0.22311 | -0.1485 | 0.0304 | 1.00E-06 | -0.0100503 | 0.0377392 | 0.79 |
| IL-17RD | rs62621196 | 6 | 149770067 | T | C | 0.00644632 | 21.40439041 | 0.04896 | 0.2631 | 0.0576 | 4.90E-06 | -0.0202027 | 0.0489806 | 0.68 |
| IL-17RD | rs6882565 | 5 | 81356421 | A | G | 0.007083643 | 23.53565765 | 0.28599 | 0.1317 | 0.027 | 1.02E-06 | -0.0100503 | 0.0197037 | 0.61 |

IL, interleukin; IL-17RA, IL-17 receptor A; SNP, single nucleotide polymorphism; Chr, chromosome; EAF, effect allele frequency; SE, standard error; Beta, effect size (log(OR) scale) estimated with revenue for the alternative allele; P value, p-value from revenue.
